# Evaluation of the mechanical properties and clinical application of nickel–titanium shape memory alloy scaphoid arc nail

**DOI:** 10.1002/elsc.202000055

**Published:** 2021-03-09

**Authors:** Muguo Song, Yongyue Su, Chuan Li, Yongqing Xu

**Affiliations:** ^1^ Department of Orthopaedics 920th Hospital of Joint Logistics Support Force Kunming Yunnan P. R. China

**Keywords:** arc nail, mechanical properties, Ni‐Ti memory alloy, scaphoid

## Abstract

To investigate the mechanical and biomechanical properties of nickel–titanium (Ni–Ti) shape memory alloy scaphoid arc nail (NT‐SAN) fixator as well as study the surgical method of treating carpal scaphoid fractures and evaluate its clinical efficacy. (1) Static and dynamic bending tests with embedded axial bending fixture were conducted to study the mechanical properties. (2) To evaluate biomechanical strength and fatigue, 32 scaphoid samples were classified into four groups to perform the fixation rigidity test: intramedullary Kirschner fixation (group A), Kirschner straddle nail fixation (group B), screw nail fixation (group C), and NT‐SAN fixation (group D). Next, 24 scaphoid waist fracture models were classified to conduct fatigue experiments as follows: Kirschner straddle nail fixation (group E), screw nail fixation (group F), and NT‐SAN fixation (group G). (3) The Krimmer score chart was used for clinical evaluations. (1) NT‐SAN showed excellent mechanical performance and a long lifespan. (2) NT‐SAN was fixated with a strong intensity and an anti‐fatigue outcome. (3) Ninety‐eight interviewed patients were satisfied with the therapeutic effects of the arc nail (satisfaction rate: 95.92%). The designed strength and hardness of NT‐SAN corresponded with the anatomical characteristics of the scaphoid, and the designed mechanical properties met the biomechanical requirements of a scaphoid fracture. The fatigue strength can meet the requirements of bone healing after the scaphoid fracture. Clinical trials on NT‐SAN scaphoid fracture treatment have shown that the surgery is simple and the clinical results are satisfactory. The therapeutic level of NT‐SAN is III; thus, it is worth promoting.

**Abbreviations**
group A: intramedullary Kirschner fixation
group B: Kirschner straddle nail fixation
group C: screw nail fixation
group D: NT‐SAN fixation
group E: Kirschner straddle nail fixation
group F: screw nail fixation
group G: NT‐SAN fixation
Ni–Ti: nickel–titanium
NT‐SAN: Ni‐Ti shape memory alloy scaphoid arc nail
NT‐SAN: Ni–Ti shape memory alloy scaphoid arc nail

## 1 INTRODUCTION

The scaphoid is a bridge between the proximal carpal and proximal carpus, which plays an important role in maintaining the stability and power transmission of the wrist; a scaphoid fracture may result in the dysfunction of the entire wrist [1]. Among wrist injuries, the scaphoid fracture is the second most common fracture following the distal radius fracture, accounting for approximately 80% of all carpal fractures. The clinical treatment and research of scaphoid fracture is the focus of the current studies [2, 3, 4]

Scaphoid fractures, especially those in the waist or proximal fractures, are prone to delayed healing, nonunion, or ischemic necrosis [4, 5]. The early diagnosis and treatment of carpal scaphoid fractures have been gradually gaining attention; moreover, internal fixation devices and surgical methods have been greatly improved. However, approximately 10%–30% of patients have experienced postoperative carpal scaphoid nonunion, osteonecrosis, and traumatic arthritis, which affect wrist function and require reoperation. For unstable fractures, most doctors advocate for surgical treatment [6, 7]. The main scaphoid fracture fixation methods include the use of Kirschner wires, Herbert nails, absorbable screws, and hollow screws [8, 9, 10]. The use of Kirschner wires to fix the scaphoid is inexpensive and easy to operate, and the tail frequently remains in the skin and is easy to remove; however, its disadvantages include intraoperative static pressure and postoperative pin track infection [8, 11]. The surgical approach and material structure of the Herbert screw have been continuously improved; therefore, Herbert screw fixation treatment of scaphoid fractures shows a good outcome, but challenges such as nonunion and fixation instability still exist, which may damage the surrounding normal articular surface when it is used to treat more serious scaphoid fractures such as unstable or broken ones [12, 13, 14]. The learning curve of Herbert screw fixation is longer; thus, it requires more clinical experience and high‐quality medical equipment [15]. The fixed strength of absorbable and hollow screw fixation was significantly weaker than that of the Herbert screw. The strength and antitorsional intensity of absorbable screws are low and weak, respectively, and they are prone to foreign body reactions. Moreover, the failing pressure between the fracture surfaces causes gaps between the fracture surfaces, which increases the probability of nonunion [15, 16, 17, 18].

**PRACTICAL APPLICATION**

This study demonstrated that the design of Ni‐Ti shape memory alloy scaphoid arc nail (NT‐SAN) in line is corresponding with the anatomical characteristics of the scaphoid, and scaphoid will be less damaged when NT‐SAN is used for navicular fracture fixation. In addition, the fixation strength can meet the biomechanical requirements of scaphoid fracture, and the fatigue strength can meet the need of bone healing after scaphoid fracture. More importantly, using arc nail fixator treatment to treat scaphoid fractures is simple and practical, and it plays a reliable role in the compression and good clinical efficacy.

Several domestic and overseas research results have revealed that the nickel–titanium (Ni–Ti) memory alloy has many qualities including excellent histocompatibility, biological degeneration, high strength, low specific gravity, low magnetism, wear resistance, corrosion resistance, fatigue resistance, and nontoxicity [19]. The Ni–Ti alloy shows the best comprehensive performance among memory alloys. Furthermore, among other advantages, it shows excellent shape memory effects, superelasticity, stable mechanical properties, good corrosion resistance, and good biocompatibility [20, 21]. Therefore, choosing the plate as the main structure can reduce the “stress shielding” effect of the bone and the damage causing arterial diseases [22]. The Ni–Ti alloy application of the arc nail materials can provide full play to its low elastic modulus, corrosion resistance, high specific strength, and good biocompatibility. Under memory biomechanics, the blood vessel ingrowth in the fracture is prolonged, which is favorable to fracture healing, and the fixation and connection of the scaphoid are intensified and tightened to provide better clinical results. Considering this, this is the first study to design the Ni–Ti memory alloy scaphoid arc fixator for scaphoid fractures, and analyze the mechanical and biomechanical properties of the fixator. Subsequently, clinical effects were demonstrated and evaluated in combination with a long‐term follow‐up. Finally, factors that influence the clinical effects were analyzed, and the surgical procedure and precautions were summarized.

## 2 MATERIALS AND METHODS

### 2.1 Ni–Ti shape memory alloy scaphoid arc nail (NT‐SAN) design

**FIGURE 1:**
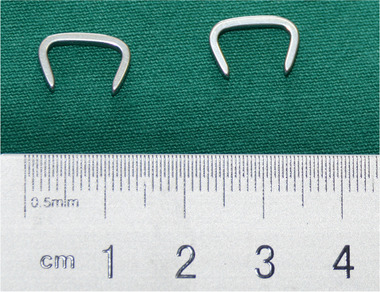
Physical map of the scaphoid screw fixation

According to the anatomical shape and measurements of the scaphoid [23, 24] and to resolve the challenges of instability, infection, and nonunion of scaphoid fracture treatment, the NT‐SAN was designed with a 1.5‐mm thick Ni–Ti shape memory alloy plate (nail length × plate width × plate thickness: 20.0 mm × 2.0 mm × 1.5 mm). It consists of an arc‐shaped body and two fixed arms; each arm and body was fixed at an angle of 70° with a one‐way memory design. The Young's modulus of Ni–Ti was 70–98 GPa. Deformation and recovery temperatures were 0–4°C and 35–40°C, respectively. There is an arc‐shaped nail physical map (Figure 1).

The alloy composition was represented by mass as follows: Ni (54.5%–57.0%), carbon (≤0.050%), cobalt (≤0.050%), copper (≤0.010%), chromium (≤0.010%), hydrogen (≤0.005%), iron (≤0.050%), columbium (≤0.025%), nitrogen + oxygen (≤0.050%), and Ti.

**FIGURE 2:**
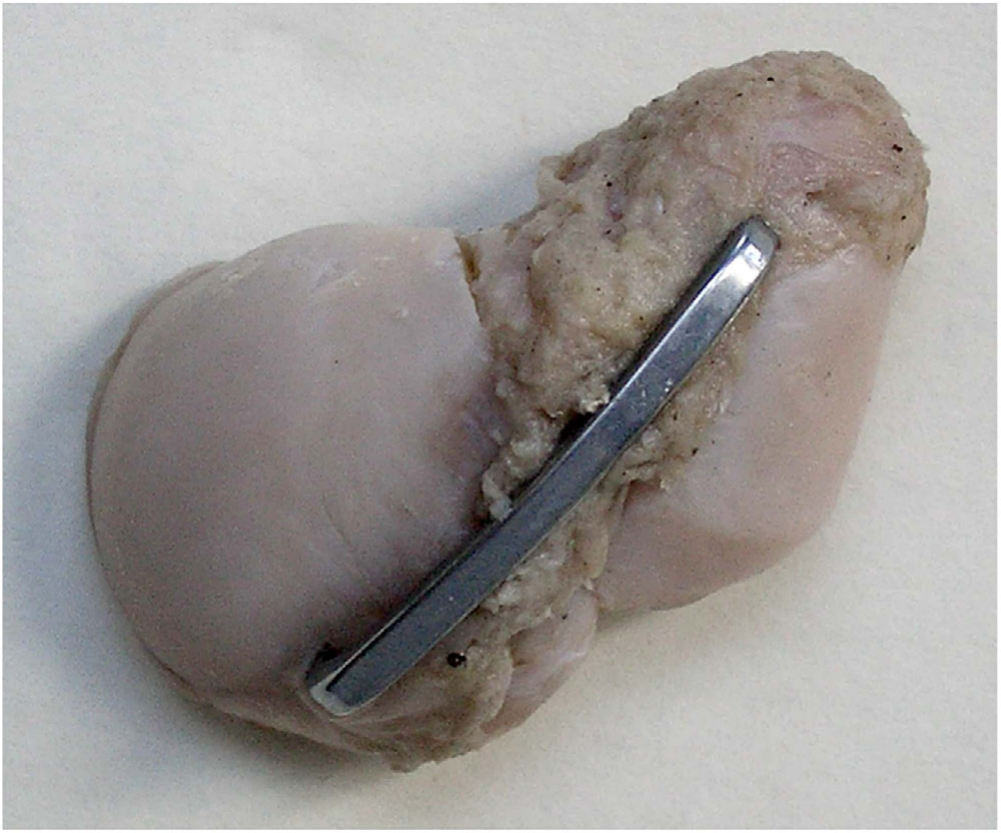
Diagram of fixing the carpal scaphoid fracture using NT‐SAN

According to the “memory principle” of the Ni–Ti memory alloy and the fact that the deformation temperature NT‐SAN was easy to maintain, the two fixed arms were stretched and inserted in the navicular scaphoid and body. When the temperature recovered, the restoring force of NT‐SAN fixed arm returned to the mother phase, polymerizing the distal fractures of the scaphoid into one (Figure 2).

### 2.2 Mechanical properties test

**FIGURE 3:**
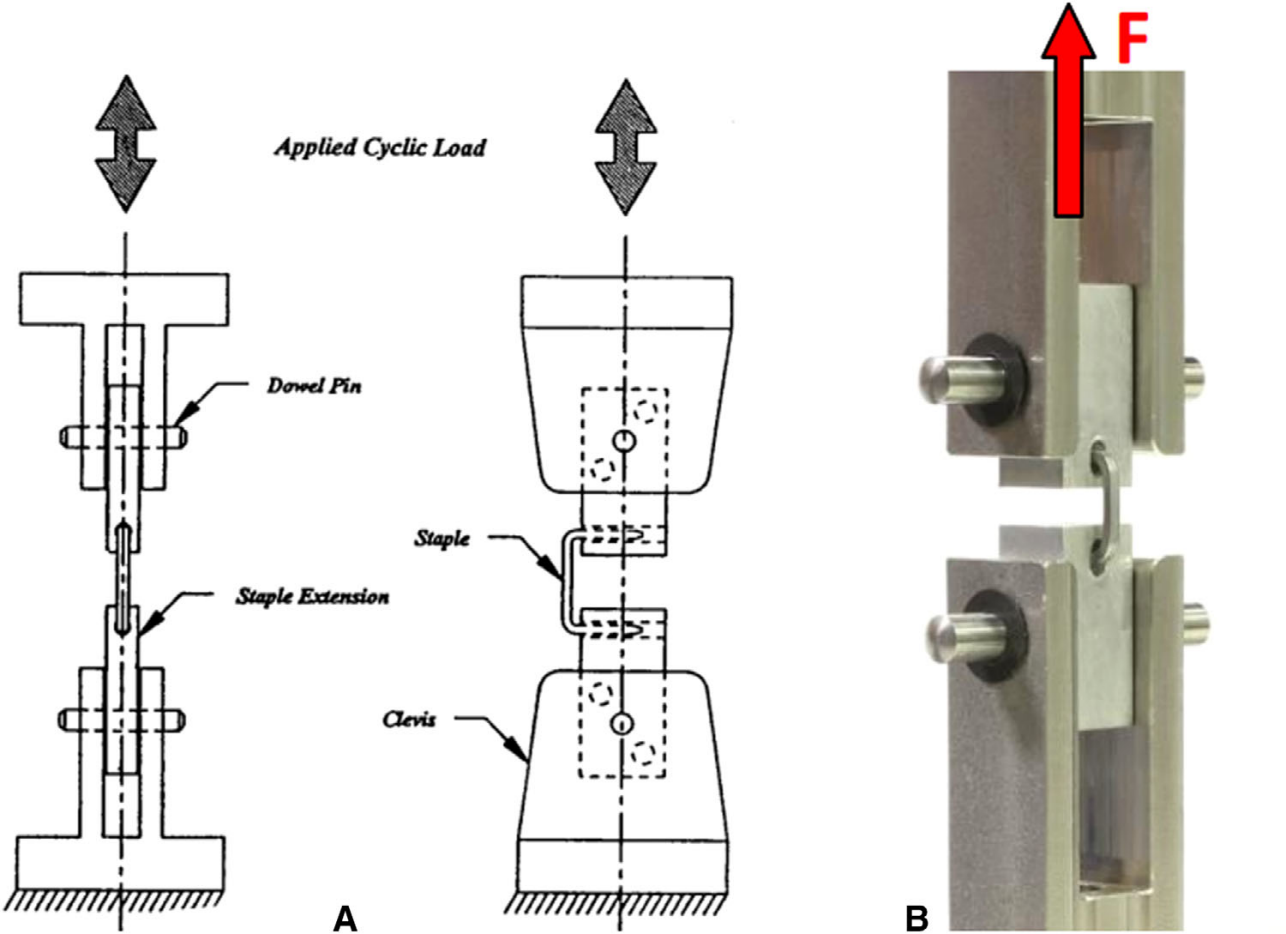
(A) Schematic diagrams of tensile bending using static tests; (B) Schematic diagrams of tensile bending using dynamic tests

In the static tests, the distance between the load and central axes of the bone nail of each specimen was measured. The load offset of the stapler inserted in the aluminum extension was 7.10 mm ± 0.10 mm and the geometric active length of the implant was 10.5 mm, which was the corresponding distance between the legs (beam, extended distance). Furthermore, the UHU Endfest® (UHU GmbH, Germany) filling agents were used to embed staple legs. In dynamic tests, the axial tension speed of the tension bending test was 10 mm/min, and the test was stopped when the arc nail broke. All tests were performed in distilled water at 37°C. The specimen was mounted on an embedded axial bending fixture (Figure 3A and B).

### 2.3 Biomechanical performance testing

The arc nail fatigue and strength tests required 56 adult upper limb specimens soaked for >1 month in 10% formaldehyde, which were provided by the Department of Anatomy, Kunming Medical College. Before the experiment, X‐ray films were used to remove the carpal bone lesions and the scaphoid was dissected. The following materials were obtained: 10 pieces of NT‐SAN (Lanzhou Ximai Memory Alloy Co., Ltd.), 1.0‐, 1.5‐, 2.0‐, and 2.5‐mm Kirschner wires and a 2.5‐mm diameter screw (Sunan Medical Equipment Manufacturing Co., Ltd.), self‐curing denture water and powder (Shanghai Medical Devices Co., Ltd. Dental Materials Factory), and Shimadzu AG‐1 50‐kN material testing system (provided by Biomedical Materials Testing Laboratory of Wood Science and Engineering Experimental Center, Southwest Forestry College).

Preparation of specimens for strength experiments:

To observe the in vitro simulation of scaphoid fracture on 32 upper limb specimens, specimens were randomly divided into four groups (n = 8) for fracture reduction and internal fixation as follows: 2.5‐mm Kirschner wire intramedullary fixation (group A), 2.0‐mm Kirschner wire stapling fixation (group B), screw fixation (group C), and NT‐SAN fixation (group D). After the classification, the self‐curing denture powder was used to embed the scaphoid nodule and body. It should be noted that the NT‐SAN body, Kirschner wire, screw, and fracture line were reserved.

Preparation of specimens for fatigue experiments:

Twenty‐four adult upper limb specimens were selected, soft tissue specimens were removed, and the integrities of the interosseous membrane and carpal transverse ligament were retained. Limbs were randomly divided into three groups (n = 8) for the reduction and internal fixation simulation of the scaphoid fracture: Kirschner wire stapler fixation (group E), screw fixation (group F), and NT‐SAN fixation (group G). Subsequently, the limbs were respectively entrapped with self‐curing denture powder at the distal ulna, proximal ulna, and metacarpal bones.

Strength test:

To perform the strength test, the A–D group specimens on the AG‐1 tester were fixed at 37°C. Continuous traction was set at the speed of 1 mm/min, and the AG‐1 tester recorded the displacement and traction value. The traction force required for 1‐mm and 2‐mm separation in the fracture space of each group was recorded, and the fixation strength of each group was evaluated.

Fatigue test:

For the fatigue test, the 98‐N vertical loads on the wrist joint of the E–G groups were vertically compressed to simulate wrist flexion and dorsiflexion movements; the range of motion was palm flexion 5°–dorsiflexion 30° [25, 26], moving 2000 times [24]. Subsequently, 1‐mm Kirschner wires were fixed in the distal and proximal scaphoid fractures as markers to measure fracture displacement. Electronic calipers (precision: 0.02 mm) were used to measure the distance between markers when the movements were 100, 500, 1500, and 2000 times. Simultaneously, the number of “steps” of the fractures (≤1 mm [I], l–2 mm [II], ≥2 mm [III]) and displacement were measured.

### 2.4 Statistical methods

SPSS18.0 statistical package was used for analysis. Data were expressed as mean ± standard deviation(s). Measured data were analyzed using the independent sample t test, and count data were analyzed using two independent samples in nonparametric tests. *P*‐value of <0.05 was considered statistically significant.

### 2.5 Clinical evaluation methods

There were 98 patients, including 89 men and nine women aged 18–55 years. All patients had closed scaphoid fractures, and the injury time was 1.5–25 days. The fracture was classified according to the Herbert classification: type A, 52 patients with stable fractures; type B, 30 patients with unstable fractures; type C, 16 patients who showed delayed healing; and type D, 0 patients who showed nonunion. The range of wrist joint function was graded according to the Krimmer score scale. Wrist function was graded according to the Krimmer score scale [23, 27]. Evaluation parameters included activity range, pain, grip strength, visual analog scale pain rating, and patient use.

### 2.6 Statement

This study was approved by the Institutional Review Board and each patient provided consent.

## 3 RESULTS

### 3.1 Mechanical properties

Static and dynamic tension/bending mode experiments were performed to analyze the mechanical properties of the NT‐SAN fixator on musculoskeletal fixation. The results of static and dynamic tests are shown in Tables 1 and 2.

**TABLE 1:** Results of static combined tension and bending test

Specimen	Yield moment (Nmm)	Yield displacement (mm)	Ultimate load (N)	Ultimate displacement (mm)	Stiffness (N/mm)
1.1	208	1.2	1.659	6.9	30.9
1.2	212	1.2	1.769	8.4	32.6
1.3	201	1.2	2.060	6.8	29.5
1.4	210	1.3	1.436	7.5	29.3
1.5	195	1.2	1.782	8.1	28.8
Mean	205	1.2	1.741	7.5	30.2
Std Dev.	7	0.1	2.26	0.7	1.5

**TABLE 2:** Results of dynamic combined tension and bending test

Specimen	Min moment (Nmm)	Max. moment (mm)	Cycles	Results
1.6	40	400	6233	Fracture
1.7	30	300	8190	Fracture
1.8	20	200	21790	Fracture
1.9	10	100	1000000	No fracture
1.10	15	150	58956	Fracture

The average moment of the NT‐SAN fixation was 205 Nmm (standard deviation: 7) when the average yield displacement was 1.2 mm (standard deviation: 0.0) in the static tension bending test. The maximum cyclic moment in the dynamic tension bending test was 100 Nm, and the maximum cycle time was 1000000. These values indicate that the memory alloy staple fixation had excellent mechanical properties for musculoskeletal fixation, which can solve challenges including instability, susceptibility to infection, and nonunion. To further meet the clinical application, the biomechanical performance was verified. The results of the strength and fatigue tests are shown in Tables 3, 4, 5.

**TABLE 3:** The traction force at 1 and 2 mm interfractmentary displacements (n = 8, N, $\bar{x}$ ± s)

	The traction force	
Group	1 mm	2 mm
A	15.18 ± 3.55*	20.28 ± 12.09*
B	36.04 ± 4.30*	75.95 ± 47.64*
C	64.84 ± 11.62	120.91 ± 26.68
D	65.84 ± 12.22	130.21 ± 31.55

* Compared with Group D, *P *< 0.05.

**TABLE 4:** The separated distance of the interfractments of scaphoid (n = 8, N, $\bar{x}$ ± s)

	The traction force (N)			
Group	100	500	1500	2000
A	1.19 ± 0.23*	1.76 ± 0.37*	2.21 ± 0.33*	2.49 ± 0.32*
B	0.46 ± 0.16*	0.92 ± 0.22*	1.44 ± 0.38*	1.89 ± 0.35*
C	0.29 ± 0.08	0.37 ± 0.09	0.44 ± 0.10	0.49 ± 0.12

* Compared with Group D, *P *< 0.05.

**TABLE 5:** The distance and “step” displacements of the interfractment of scaphoid

	The traction force (N)											
Group	100			500			1500			2000		
	I	II	III	I	II	III	I	II	III	I	II	III
E	5	3	0	1	6	1	0	5	3	0	5	3
F	7	1	0	5	3	0	1	6	1	1	4	3
G	8	0	0	8	0	0	7	1	0	6	2	0

The traction forces at 1‐ and 2‐mm interfractmentary displacements are shown in Table 3. A statistically significant (*P* < 0.05) difference was observed between group D and groups A and B, whereas no significant (*P* > 0.05) difference was observed between groups D and C. The separated distance of the interfractional segments of the scaphoid is shown in Table 4. Comparing group G with groups E and F, all indicators showed a significant difference (*P* < 0.05). Comparing groups G and E, each index after 500 times of wrist joint movements had a significant difference (*P* < 0.05). The distance and “step” displacements of the interfractional segments of the scaphoid are shown in Table 5. Comparing groups G amd F, each index after 1500 times of wrist joint movements showed a significant difference (*P* < 0.05). This is primarily attributable to the fact that the restoring force of the fixed NT‐SAN arm can polymerize the scaphoid fracture to one; thus, the body can block the slip of the scaphoid fracture [28, 29].

### 3.2 Clinical application results

**FIGURE 4:**
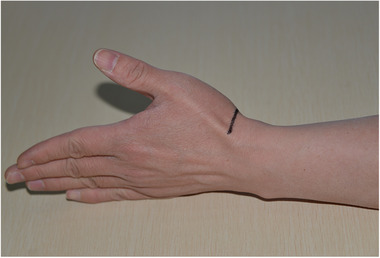
Surgical incision

**FIGURE 5:**
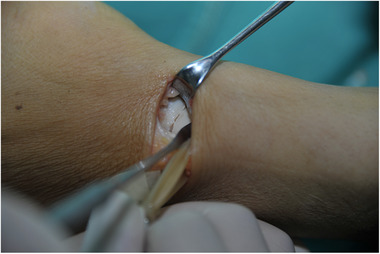
Scaphoid fracture operated in a layer‐by‐layer manner

**FIGURE 6:**
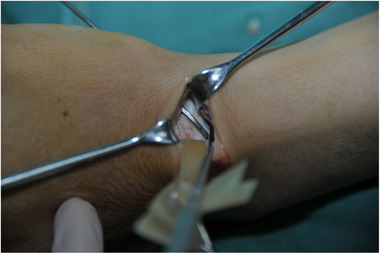
Fixed scaphoid fracture

General anesthesia or brachial plexus block anesthesia. At the proximal side of the wrist palm with wrist stripe level, a transverse incision was made between the radial dorsal carpal flexor and flexor hallucis longus tendon (Figure 4). After freeing the subcutaneous tissue, the two tendons were respectively retracted to the ulnar and radial sides, and then exposed and retracted the radial artery, vein, and nerves. Scaphoid fractures were revealed in a layer‐by‐layer fashion (Figure 5). Next, a small amount of soft tissue was stripped at the proximal and distal ends of the fracture; after the scaphoid fracture trial was reset, a 2.0‐mm Kirschner wire was used to design the screw channel in the proximal and distal ends. Subsequently, a vascular clamp was used to slightly expand the nail road, distracting the two nails to the ends after selecting the appropriate type of memory alloy nail foot holder and placing it in ice water, and the small hammer was beaten to ensure that the nail foot was fully embedded. The scaphoid was soaked in warm water (45°), pressed, and fixed (Figure 6); if there were small free bones in the fracture, they were roughly reset and compressed under the scaphoid fixator. For patients with more severe fragmentation and fracture displacement, an assisted Kirschner wire can be used to fix their fractures. Plaster immobilization is not necessary after surgery.

**FIGURE 7:**
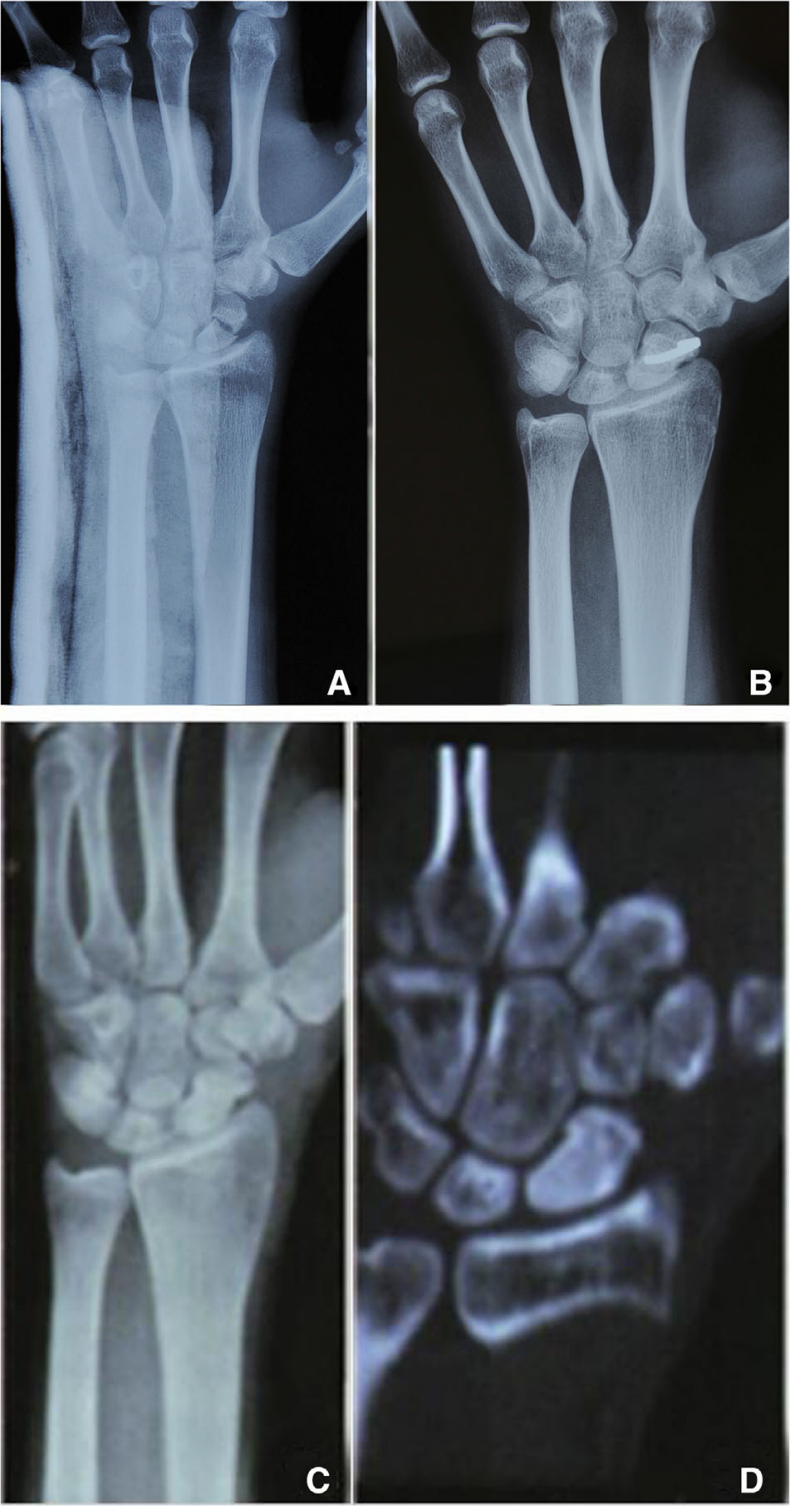
(A) Typical preoperative and postoperative cases; (B) Typical preoperative and postoperative cases; (C) Typical preoperative and postoperative cases; (D) Typical preoperative and postoperative cases

Among 98 patients, the fractures of 68 patients were fixed with an arc stapler (protocol 1), 14 patients were underwent Kirschner wire fixation, and 16 patients underwent autogenous bone graft. Postoperative wound healing period was period I, and the follow‐up period ranged from 8 to 24 months. All fractures healed without infection, nonunion, traumatic arthritis, and other complications. The Krimmer scores were in the range of 78–98, and the average was 92, with 89, 5, and 4, excellent, good, and satisfactory cases, respectively. The rate of excellent outcomes was 95.92%. Typical cases were compared before and after surgery (Figure 7A–D).

Figure 7A and B are preoperative and postoperative x‐ray films of a patient with left scaphoid lumbar transverse fractures; Figure 7C and D are the X‐ray and computed tomography images of the removed scaphoid lumbar with curved nails. It is apparent that the scaphoid healed well and achieved the expected surgery outcome. Wrist flexion, dorsiflexion activity, and grip strength of 68 patients who used arc staplers were measured preoperatively (control group) and postoperatively (treatment group). Measurement results are shown in Table 6.

**TABLE 6:** Comparison of wrist flexion, dorsiflexion activity and grip strength in patients before and after treatment ($\bar{x}$ ± s)

	Case	Dorsiflexion activity (level)	Wrist flexion (M)	Local pain (point)	Grip strength (N)
Before operation	68	39.97 ± 4.58	31.08 ± 3.94	11.06 ± 1.14	19.37 ± 21.35
After operation	68	67.12 ± 6.17	49.12 ± 5.07	14.08 ± 1.53	39.84 ± 19.87

As shown in Table 6, irrespective of left or right hand, the preoperative and postoperative wrist flexion, dorsiflexion activity, and grip strength showed significant differences; that is, wrist flexion, dorsiflexion activity, and grip strength increased postoperatively. Thus, the clinical arc nail fixation device could help patients restore wrist function.

## 4 DISCUSSION

Whether the mechanical properties of NT‐SAN can meet the requirements is an important reference for its the clinical application. The NT‐SAN fixation was compared with the Kirschner wire and Kirschner wire stapling fixation [30]. The difference was statistically significant, and the NT‐SAN showed greater fixation strength than the Kirschner wire fixation [31]. Comparing the NT‐SAN fixation with screw fixation, the difference in fixation intensity of the two groups showed no statistical significance. However, when the wrist performed >500 cycle activities, the stability of the NT‐SAN fixation, screw fixation, and Kirschner wire showed statistically significant difference. Simultaneously, the traditional screw and Kirschner wire are highly ordered austenitic metallographic structures; therefore, the pressure of these materials was passive. The Ni–Ti memory alloy can generate continual fixation under body temperature, and its active compression will enhance the effectiveness of the fixation.

The NT‐SAN corresponds with the anatomical characteristics of the scaphoid; thus, NT‐SAN can cover up for the disadvantages of internal fixation devices. Furthermore, the surgery procedure is simple. The NT‐SAN should be placed in 0–4°C saline, then two fixed arms are extended and NT‐SAN is directly placed in the scaphoid nodules and dorsal non‐articular surface of the body. When using saline at 40°C to heat the NT‐SAN body, and the arm is fixed to restore the mother phase of the restoring force, the force can polymerize both ends of the scaphoid fracture to one. Simultaneously, the NT‐SAN body can prevent the fracture from slipping to the dorsal side, which corresponds with the tension band principle; thus, improving the healing rate of the scaphoid fracture.

Furthermore, the NT‐SAN body is close to the carpal surface; thus, tendon rupture complications due to tendon slip can be reduced. The arc fixed nail of the arc nail fixator and its special design is more conducive to bone grafting, pressure reduction, and fixation. Therefore, no external fixation is required, direct vision is reset, operation is easy, the operation period is shortened, and the risk of infection is reduced. Excessive wrist dorsiflexion and radial deviation, wrist line non‐load master, and passive functional exercise should be primarily avoided until the bone heals. This is also an advantage of the proposed fixator compared with the Herbert nail hollow screw.

However, the Ni–Ti shape memory alloy also has certain drawbacks. Although a protective layer of Ti‐based oxide exists as an effective barrier to the release of Ni ions, the high concentration of Ni in the alloy increases the risk of allergic reactions [32]. Therefore, researchers focus on the development of Ni‐free Ti‐based shape memory alloys that are composed of nontoxic elements [33]. Furthermore, different processing methods have different effects on the microstructure of the Ni–Ti shape memory alloy, further affecting its shape memory properties and deformation behavior, and thereby limiting its application in the medical field. The plastic forming ability of the Ni–Ti shape memory alloy has several specific requirements of materials, stress, and temperature. It is even more challenging to produce good, superelastic thin‐walled pipes with suitable phase transition temperatures for medical use. In recent years, several scholars have changed the mold structure, billet size, heating method, and temperature to obtain Ni–Ti shape memory alloy pipes for medical use with good performance [34].

## 5 CONCLUDING REMARKS

The NT‐SAN design corresponds with the anatomical characteristics of scaphoid fractures. Surgery is simple, and the scaphoid experiences less damage when the NT‐SAN is used for navicular fracture fixation. The fixation and fatigue strengths can meet the biomechanical requirements and the requirements of bone healing after the scaphoid fracture, respectively. Using the arc nail fixator to treat scaphoid fractures is simple and practical, and it plays a reliable role in compression with good clinical efficacy.

## CONFLICT OF INTEREST

The authors have declared no conflict of interest.

## FUNDING

This work was supported by the Yunnan Provincial Zhong Shizhen Academician Work Station Fund (Grant number: 2015IC30).

## AUTHORS’ CONTRIBUTIONS

Songmu Guo and Xuyong Qing designed the experiments, and Suyong Yue and Li performed the experiments and analyzed the results. Songmu Guo wrote the manuscript. Xuyong Qing revised the manuscript. All authors approved the final manuscript. Muguo Song, Yongyue Su and Chuan Li are as equal first coauthors.

## Data Availability

The data that support the findings of this study are available from the corresponding author upon reasonable request.
